# Severe Acute Respiratory Syndrome Coronavirus 2−Specific Antibody Responses in Coronavirus Disease Patients

**DOI:** 10.3201/eid2607.200841

**Published:** 2020-07

**Authors:** Nisreen M.A. Okba, Marcel A. Müller, Wentao Li, Chunyan Wang, Corine H. GeurtsvanKessel, Victor M. Corman, Mart M. Lamers, Reina S. Sikkema, Erwin de Bruin, Felicity D. Chandler, Yazdan Yazdanpanah, Quentin Le Hingrat, Diane Descamps, Nadhira Houhou-Fidouh, Chantal B.E.M. Reusken, Berend-Jan Bosch, Christian Drosten, Marion P.G. Koopmans, Bart L. Haagmans

**Affiliations:** Erasmus Medical Center, Rotterdam, the Netherlands (N.M.A. Okba, C.H. GeurtsvanKessel, M.M. Lamers, R.S. Sikkema, E. deBruin, F.D. Chandler, C.B.E.M. Reusken, M.P.G. Koopmans, B.L. Haagmans);; Charité-Universitätsmedizin Berlin, Berlin, Germany (M.A. Müller, V.M. Corman, C. Drosten);; German Centre for Infection Research, Berlin (M.A. Müller, V.M. Corman, C. Drosten);; Utrecht University, Utrecht, the Netherlands (W. Li, C. Wang, B.-J. Bosch);; Université de Paris, Paris, France (Y. Yazdanpanah, Q. Le Hingrat, D. Descamps);; Hôpital Bichat-Claude Bernard, Paris (Y. Yazdanpanah, Q. Le Hingrat, D. Descamps, N. Houhou-Fidouh);; RIVM, Bilthoven, the Netherlands (C.B.E.M. Reusken)

**Keywords:** Severe acute respiratory syndrome coronavirus 2, SARS-CoV-2, coronavirus, viruses, human coronavirus, HCoV, coronavirus disease 2019, COVID-19, antibodies, serologic analysis, spike protein, receptor-binding domain, RBD, nucleocapsid protein, neutralization, ELISA, respiratory infections, zoonoses

## Abstract

A new coronavirus, severe acute respiratory syndrome coronavirus 2 (SARS-CoV-2), has recently emerged to cause a human pandemic. Although molecular diagnostic tests were rapidly developed, serologic assays are still lacking, yet urgently needed. Validated serologic assays are needed for contact tracing, identifying the viral reservoir, and epidemiologic studies. We developed serologic assays for detection of SARS-CoV-2 neutralizing, spike protein–specific, and nucleocapsid-specific antibodies. Using serum samples from patients with PCR-confirmed SARS-CoV-2 infections, other coronaviruses, or other respiratory pathogenic infections, we validated and tested various antigens in different in-house and commercial ELISAs. We demonstrated that most PCR-confirmed SARS-CoV-2–infected persons seroconverted by 2 weeks after disease onset. We found that commercial S1 IgG or IgA ELISAs were of lower specificity, and sensitivity varied between the 2 assays; the IgA ELISA showed higher sensitivity. Overall, the validated assays described can be instrumental for detection of SARS-CoV-2–specific antibodies for diagnostic, seroepidemiologic, and vaccine evaluation studies.

In December 2019, a new coronavirus emerged in China and caused an acute respiratory disease now known as coronavirus disease 2019 (COVID-19) (*1*). The virus was identified to be a betacoronavirus related to severe acute respiratory syndrome coronavirus (SARS-CoV) and thus was named SARS-CoV-2 (*2*). In <2 decades, this virus is the third known coronavirus to cross the species barrier and cause severe respiratory infections in humans after SARS-CoV in 2003 and Middle East respiratory syndrome coronavirus (MERS-CoV) in 2012, yet with unprecedented spread compared with the earlier 2 viruses.

Because of the rapid increase in number of cases and uncontrolled and vast spread worldwide, the World Health Organization has declared SARS-CoV-2 a pandemic. As of March 14, 2020, the virus had infected >130,000 persons in 122 countries, 3.7% of whom had died. (*3*). Rapid identification of the etiology and sharing of the genetic sequence of the virus, followed by international collaborative efforts initiated because of emergence of SARS-CoV-2, has led to rapid availability of real-time PCR diagnostic assays that support case ascertainment and tracking of the outbreak (*4*). Availability of these assays has helped in patient detection and efforts to contain the virus. However, validated serologic assays are still lacking and are urgently needed.

Validated serologic assays are crucial for patient contact tracing, identifying the viral reservoir hosts, and epidemiologic studies. Epidemiologic studies are urgently needed to help uncover the burden of disease, in particular the rate of asymptomatic infections, and to get better estimates on illness and death. In addition, these epidemiologic studies can help identify the extent of virus spread in households, communities, and specific settings, which could help guide control measures. Serologic assays are also needed for evaluation of results of vaccine trials and development of therapeutic antibodies.

Among the 4 coronavirus structural proteins, the spike (S) and nucleocapsid (N) proteins are the main immunogens (*5*). We describe development of serologic assays for detection of virus neutralizing antibodies and antibodies to the N protein and various S protein domains, including the S1 subunit, and the receptor-binding domain (RBD) of SARS-CoV-2 in an ELISA format. Using a well-characterized cohort of serum samples from PCR-confirmed SARS-CoV-2 and patients PCR-confirmed to be infected with seasonal coronaviruses and other respiratory pathogens, we validated and tested various antigens in different platforms developed in-house, as well as a commercial platform.

## Materials and Methods

### Serum Samples

#### Erasmus Medical Center Samples

We used serum samples (n = 10) collected from 3 PCR-confirmed patients: 2 with mild COVID-19 and 1 with severe COVID-19 (Table 1) from France in accordance with local ethics approvals (F.-X. Lescure et al., unpub. data, https://doi.org/10.1101/2020.03.11.987958). For assay validation, we used samples obtained from persons who had PCR-diagnosed infections with human coronaviruses (HCoV-229E, NL63, or OC43), SARS-CoV, MERS-CoV, or other respiratory viruses (Table 1) as reported (*6*). We also included samples from patients who had recent infections with cytomegalovirus, Epstein-Barr virus, or *Mycoplasma pneumoniae* because these pathogens have a higher likelihood of causing false-positive results. As negative controls, we used serum samples from 45 healthy blood donors (Sanquin Blood Bank, https://www.sanquin.nl) (cohort A). We also tested serum samples from SARS patients (*7*). All samples were stored at −20°C until use. The Sanquin Blood Bank obtained written informed consent for research use of samples from blood donors. Use of serum samples from the Netherlands was approved by the local medical ethics committee (approval no. 2014–414). 

**Table 1:** Cohorts used to validate specificity and sensitivity of assays for SARS-CoV-2*

Cohort	Country	Sample source	Infection	No. samples	Postdiagnosis range or time
A	The Netherlands	Healthy blood donors (negative cohort)	NA	45	NA
B	The Netherlands	Non-CoV respiratory infections†	Adenovirus	5	2–4 wk
			Bocavirus	2	2–4 wk
			Enterovirus	2	2–4 wk
			HMPV	9	2–4 wk
			Influenza A	13	2–4 wk
			Influenza B	6	2–4 wk
			Rhinovirus	9	2–4 wk
			RSV	9	2–4 wk
			PIV-1	4	2–4 wk
			PIV-3	4	2–4 wk
			*Mycoplasma pneumoniae*	1	2–4 wk
			CMV	5	2–4 wk
			EBV	7	2–4 wk
C	The Netherlands	HCoV infections†	α-CoV HCoV-229E	19	2 w–1 y
			α-CoV HCoV-NL63	18	2 w–1 y
			β-CoV HCoV-OC43	38	2 w–1 y
D	The Netherlands	Zoonotic CoV infections†	MERS-CoV	2	10,228 d
	South Korea			5	9 mo
E	Hong Kong, China	Zoonotic CoV infection†	SARS-CoV	2	>14 d
F	France	RT-PCR confirmed SARS-CoV-2 infections	Mild infection	6‡	3–27 d
			Severe infection	4§	6–31 d

*Cohorts A–E were used to test assay specificity; cohort F was used to test assay sensitivity. α-CoV, alphacoronavirus; β-CoV, betacoronavirus; CoV, coronavirus; CMV, cytomegalovirus; EBV, Epstein-Barr virus; HCoV, human coronavirus; HMPV, human metapneumovirus; MERS, Middle East respiratory syndrome; NA, not applicable; PIV, parainfluenza virus; RSV, respiratory syncytial virus; RT-PCR, reverse transcription PCR. †Cross-reactivity. ‡Samples taken from 2 patients at different time points. §Samples taken from 1 patient at different time points.

#### Berlin Samples

All serum samples (n = 31) from patients with PCR-confirmed cases of COVID-19 cases were previously analyzed by a recombinant SARS-CoV-2 S protein–based immunofluorescence test and plaque reduction neutralization (R. Wölfel et al., unpub. data, https://doi.org/10.1101/2020.03.05.20030502). We tested serum samples as part of an extended diagnostic regimen after we obtained informed written consent from patients. We obtained non–SARS-CoV-2–infected serum samples (n = 31) from the serum collection of the National Consiliary Laboratory for Coronavirus Detection at Charité–Universitätsmedizin Berlin (Berlin, Germany). Samples were collected after we obtained informed written consent. The collection contained follow-up antibody-positive serum samples from PCR-confirmed virus-infected cases: HCoV-229E (n = 4), HCoV-HKU1 (n = 3), HCoV-OC43 (n = 7), MERS-CoV (n = 3), HCoV-NL63 (n = 6), SARS-CoV (n = 3), and common cold CoV (n = 6).

### Protein Expression

We expressed the S ectodomains of SARS-CoV-2 (residues 1–1,213, strain Wuhan-Hu-1, GenBank accession no. QHD43416.1), SARS-CoV (residues 1–1,182, strain CUHK-W1, accession no. AAP13567.1), and MERS-CoV (residues 1–1262, strain EMC, accession no. YP_009047204.1) in HEK-293T cells by using a C-terminal trimerization motif, Strep-tag, and the pCAGGS expression plasmid. Likewise, we expressed the SARS-CoV-2 S1 subunit or its subdomains (S;S1, residues 1–682; S1^A^, residues 1–294; RBD, residues 329–538; accession no. QHD43416.1) in 293T cells, as described (C. Wang et al., unpub. data, https://doi.org/10.1101/2020.03.11.987958).

We produced S1 proteins of other HCoVs: HKU1 (residues 1–750), OC43 (residues 1–760), NL63 (residues 1–717), 229E (residues 1–537), SARS-CoV (residues 1–676), and MERS-CoV as described (*6*,*8*). We affinity purified all recombinant proteins from culture supernatant by using Protein-A Sepharose beads (catalog no. 17–0780–01; GE Healthcare, GE Healthcare, https://www.gehealthcare.com) or strep-tactin beads (catalog no. 2–1201–010; IBA Lifesciences, https://www.iba-lifesciences.com). We checked purity and integrity of all purified recombinant proteins by using sodium dodecyl sulfate–polyacrylamide gel electrophoresis and staining with Coomassie blue.

### Plaque Reduction Neutralization Test

We used the plaque reduction neutralization test (PRNT) as a reference for this study because neutralization assays are the standard for coronavirus serologic analysis. We tested serum samples for their neutralization capacity against SARS-CoV-2 (German isolate; GISAID ID EPI_ISL 406862; European Virus Archive Global #026V-03883) by using PRNT as described with some modifications (*9*). We 2-fold serially diluted heat-inactivated samples in Dulbecco modified Eagle medium supplemented with NaHCO_3_, HEPES buffer, penicillin, streptomycin, and 1% fetal bovine serum, starting at a dilution of 1:10 in 50 μL. We then added 50 μL of virus suspension (400 plaque-forming units) to each well and incubated at 37°C for 1 h before placing the mixtures on Vero-E6 cells. After incubation for 1 h, we washed, cells supplemented with medium, and incubated for 8 h. After incubation, we fixed the cells with 4% formaldehyde/phosphate-buffered saline (PBS) and stained the cells with polyclonal rabbit anti-SARS-CoV antibody (Sino Biological, https://www.sinobiological.com) and a secondary peroxidase-labeled goat anti-rabbit IgG (Dako, https://www.agilent.com). We developed signal by using a precipitate forming 3,3′,5,5′-tetramethylbenzidine substrate (True Blue; Kirkegaard and Perry Laboratories, https://www.seracare.com) and counted the number of infected cells per well by using an ImmunoSpot Image Analyzer (CTL Europe GmbH, https://www.immunospot.eu). The serum neutralization titer is the reciprocal of the highest dilution resulting in an infection reduction of >50% (PRNT_50_). We considered a titer >20 to be positive.

We performed the PRNT for serum samples from Germany by using Vero E6 cells, as described (R. Wölfel et al., unpub. data, https://doi.org/10.1101/2020.03.05.20030502) (*10*) and 24-well plates. Before the PRNT, we heat-inactivated patient serum samples at 56°C for 30 min. For each dilution step (in duplicate), we diluted patient serum samples in 200 μL of OptiPro serum-free medium (https://www.thermofisher.com) and mixed 1:1 with 200 μL of virus solution containing 100 PFUs. We vortexed the 400-μL serum–virus solution gently, incubated at 37°C for 1 h, and then incubated each 24-well plate with 200 μL serum–virus solution. After incubation for 1 h at 37°C, we discarded supernatants, washed cells once with PBS, and supplemented them with 1.2% microcrystalline cellulose solution in Dulbecco modified Eagle medium. After 3 days, we fixed and inactivated the plates by using a 6% formaldehyde/PBS solution and stained with crystal violet.

### ELISA

We performed anti-SARS-CoV-2 S1 IgG and IgA ELISAs by using β-versions of 2 commercial kits (EUROIMMUN Medizinische Labordiagnostika AG, https://www.euroimmun.com) and performed the assay according to the manufacturer’s protocol. We detected optical density (OD) at 450 nm and calculated a ratio of the reading of each sample to the reading of the calibrator, included in the kit, for each sample (OD ratio). Because the β-version of the kit awaits validation and marking, we determined an in-house cutoff value based on the mean background reactivity of all SARS-CoV-2–negative serum samples in the study multiplied by 3. The OD ratio was 0.9 for IgA and 0.3 for IgG.

We performed the in-house ELISAs by coating 96-well microtiter ELISA plates with in-house–produced S antigens (S or S1 of SARS-CoV-2, SARS-CoV or MERS-CoV; SARS-CoV-2 S1^A^; or RBD proteins) or SARS-CoV N protein (Sino Biological) in PBS overnight at 4°C. After blocking, we added diluted serum (diluted 1:100 or 2-fold serially diluted for titers) and incubated at 37°C for 1 h. Antigen-specific antibodies were detected by using peroxidase-labeled rabbit anti-human IgG (Dako) and 3,3′,5,5′-tetramethylbenzidine as a substrate. The absorbance of each sample was measured at 450 nm, and we set the cutoff value at 6 SD above the mean value for the negative cohort.

### S1 Protein Microarray

Serum samples were previously tested for antibodies against S1 of different coronaviruses. We used a protein microassay that has been described (*6*).

### Statistical Analysis

We analyzed the correlations between antibody responses detected by different ELISAs and those detected by PRNT, which is the standard for coronavirus serologic analysis. We used GraphPad Prism version 8 (https://www.graphpad.com) for this analysis.

## Results

**Figure 1:**
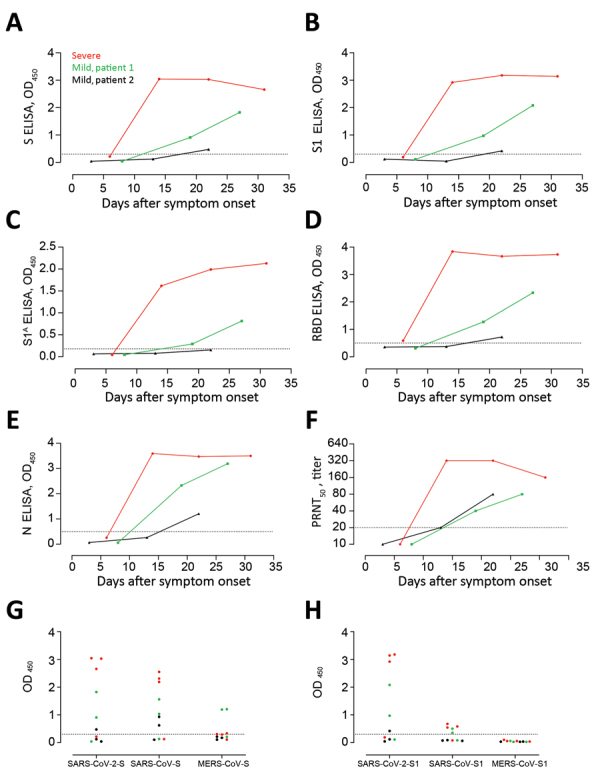
Kinetics of antibody responses against SARS-CoV-2 after infection. We tested 1 patient who had severe coronavirus disease 2019 (red) and 2 patients who had mild coronavirus disease 2019 (green and black) for antibody responses against A) S protein, B) S protein S1 subunit, C) S N-terminal (S1^A^) domain, D) RDB, and E) N protein by using ELISAs. F) Virus-neutralizing antibodies were tested by using a PRNT_50_. G, H) Reactivities of serum samples from the 3 patients at different time points against whole S (G) and S1 (H) of SARS-CoV-2, SARS-CoV, and MERS-CoV were tested by ELISAs. Dotted horizontal lines indicate ELISA cutoff values. MERS-CoV, Middle East respiratory syndrome coronavirus; N, nucleocapsid; OD, optical density; PRNT_50_, 50% plaque reduction neutralization test; RBD, receptor-binding domain; S, spike; SARS-CoV, severe acute respiratory syndrome coronavirus; SARS-CoV-2, severe acute respiratory syndrome coronavirus 2.

**Figure 2:**
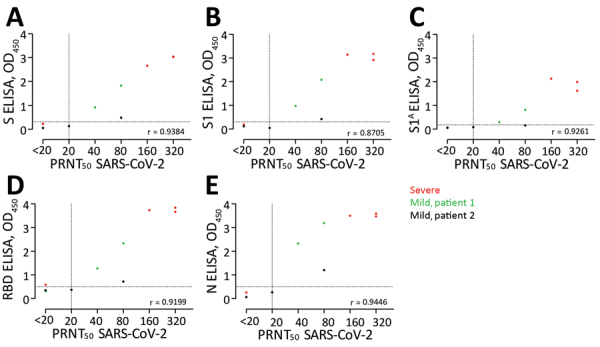
Correlations between ODs of ELISAs and PRNT results for PCR-confirmed COVID-19 patients. A) S; B) S1; C) S1^A^; D) RBD; E) N. Ten serum samples were collected 6–27 days after diagnosis from 3 COVID-19 patients in France. Dots indicate patients. Dotted horizontal lines indicate ELISA cutoff values. COVID-19, coronavirus disease 2019; N, nucleocapsid; OD, optical density; PRNT_50_, 50% plaque reduction neutralization test; RBD, receptor-binding domain; S, spike; SARS-CoV-2, severe acute respiratory syndrome coronavirus 2.

We evaluated SARS-CoV-2–specific antibody responses in severe and mild cases by using serum samples collected at different times postonset of disease from 3 PCR-confirmed COVID-19 patients from France. We tested serum samples for SARS-CoV-2–specific antibodies by using different ELISAs. After infection, all 3 patients seroconverted between days 13 and 21 after onset of disease (Figure 1), and antibodies were elicited against the SARS-CoV-2 S, S1 subunit, and RBD, but only 2/3 patients had detectable antibodies to the N-terminal (S1^A^) domain. Because the N protein of SARS-CoV-2 is 90% similar to that of SARS-CoV (Table 2), we used SARS-CoV N protein as an antigen to test for SARS-CoV-2 N protein–directed antibodies in an ELISA format. We found that antibodies were elicited against the N protein in all three patients. When tested in a PRNT, serum samples from all three patients neutralized SARS-CoV-2 infection. Antibody responses detected by different assays correlated strongly with neutralizing antibody responses (Figure 2). We observed cross-reactivity with the SARS-CoV S and S1 proteins, and to a lower extent with MERS-CoV S protein, but not with the MERS-CoV S1 protein (Figure 1, panels G, H). This finding was evident from analyzing the degree of similarity of the different coronavirus S protein domains to their corresponding SARS-CoV-2 proteins (Table 2). This analysis showed that the S2 subunit is more conserved and thus plays a role in the cross-reactivity seen when the whole S was used as antigen. Thus, S1 is more specific than S as an antigen for SARS-CoV-2 serologic diagnosis.

**Table 2:** Percentage amino acid identity of coronavirus spike and nucleocapsid proteins with SARS-CoV-2 proteins*

*Virus type*	*Virus*	*Nucleocapsid*	*S*	*S1*	*S2*	*S1^A^*	*RBD*
*Betacoronavirus*	SARS-CoV	90	77	66	90	52	73
	MERS-CoV	49	33	24	43	ND	ND
	HCoV-OC43	34	33	25	42	ND	ND
	HCoV-HKU1	34	32	25	40	ND	ND
*Alphacoronavirus*	HCoV-229E	28	30	24	35	ND	ND
	HCoV-NL63	29	28	21	36	ND	ND

*SARS-CoV-2, HCoV-OC43, MERS-CoV, HCoV-HKU1, HCoV-NL63, SARS-CoV, HCoV-229E (GenBank accession nos. NC_045512.2, NC_006213.1, NC_019843.3, NC_006577.2, NC_005831.2, NC_004718.3, and NC_002645.1). Protein sequences were aligned by using ClustalW (https://www.genome.jp/tools-bin/clustalw). RBD, receptor-binding domain; ND, not done; S, spike; S1, N-terminal subunit of the spike protein; S2, C-terminal subunit of the spike protein; S1^A^, domain A of the spike S1 subunit; SARS-CoV, severe acute respiratory syndrome coronavirus; SARS-CoV-2, severe acute respiratory syndrome coronavirus 2.

**Figure 3:**
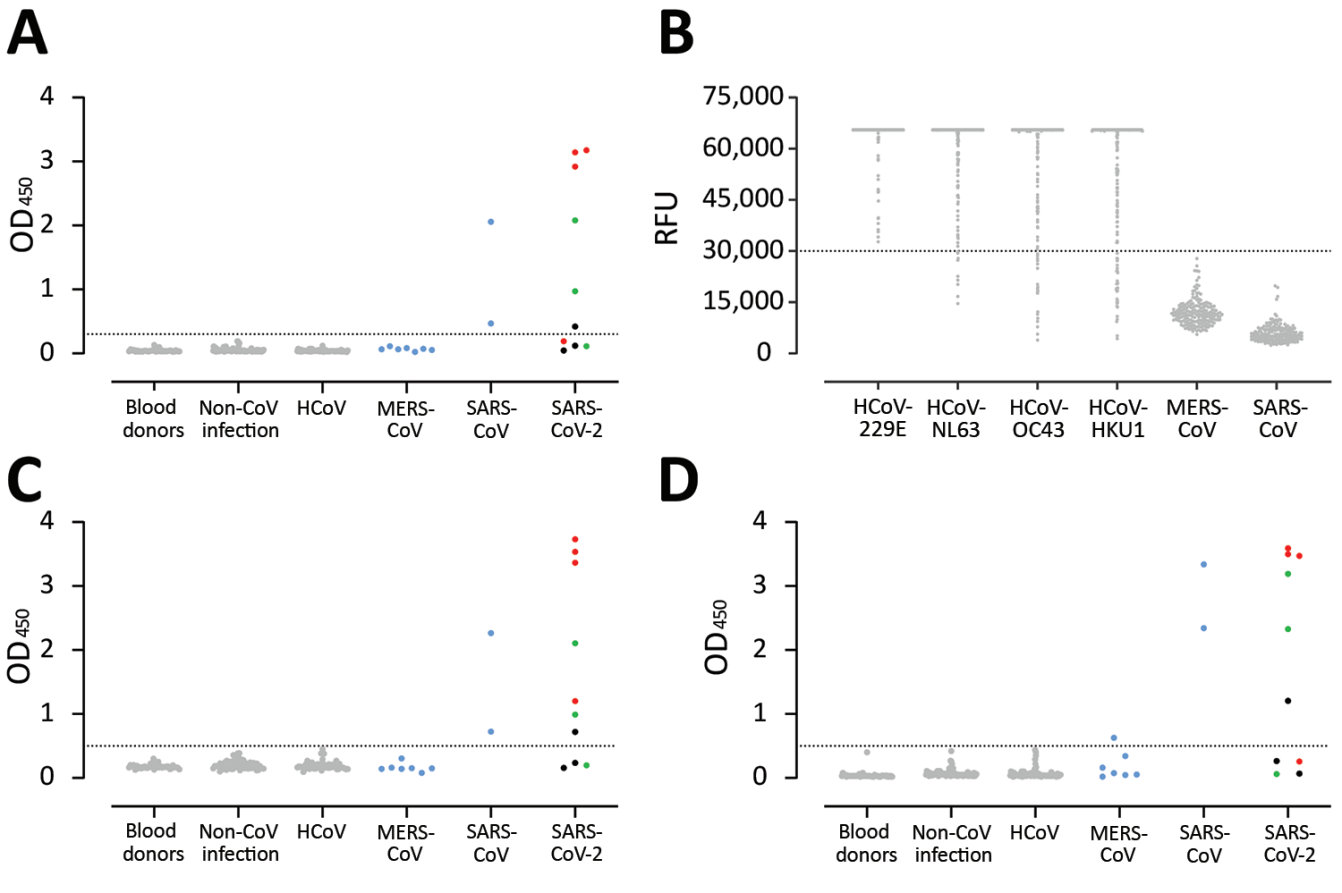
Validation of use of S1 (A, B), RBD (C), and N protein (D) ELISAs for detection of SARS-CoV-2–specific antibodies infections. Gray dots indicate specificity cohorts A–C, including healthy blood donors (n = 45), non-CoV respiratory infections (n = 76), and HCoV infections (n = 75); blue dots indicate non-SARS-CoV-2 zoonotic coronavirus infections (i.e., MERS-CoV [n = 7] and SARS-CoV [n = 2]); red dots indicate patients with severe COVID-19; and green and black dots indicate patients with mild COVID-19. Dotted horizontal lines indicate ELISA cutoff values. CoV, coronavirus; COVID-19, coronavirus disease 2019; HCoV, human coronavirus; MERS-CoV, Middle East respiratory syndrome coronavirus; N, nucleocapsid; OD, optical density; RBD, receptor-binding domain; RFU, relative fluorescence unit; S, spike; SARS-CoV, severe acute respiratory syndrome coronavirus; SARS-CoV-2; severe acute respiratory syndrome coronavirus 2.

We further assessed the specificity of the S1 assay by using cohorts A–E (Table 1), which were composed of serum samples from healthy blood donors (A), PCR-confirmed acute respiratory non-CoV infections (B), acute-phase and convalescent-phase PCR-confirmed α- and β-HCoV infections (C), PCR-confirmed MERS-CoV infections (D), and PCR-confirmed SARS-CoV infections (E). None of the serum samples from specificity cohorts A–D were reactive in our in-house S1 ELISA at the set cutoff value, indicating 100% specificity, whereas serum samples from SARS-CoV patients cross-reacted (Figure 3, panel A). The specificity of S1 as an antigen for SARS-CoV-2 serologic analysis was further supported by the fact that 87%–100% of serum samples in cohorts A–C included in this study were seropositive for endemic HCoVs (HCoV-HKU1, HCoV-OC43, HCoV-NL63, and HCoV-229E), as determined by the S1 protein microarray (Figure 3, panel B). Nonetheless, all serum samples were seronegative for SARS-CoV and MERS-CoV.

Using the same cohort, we also validated the specificity of the N protein IgG and RBD IgG ELISAs for detecting SARS-CoV-2–specific antibodies. At the set cutoff, except for serum samples from SARS-CoV patients, none of the control serum samples was positive for RBD antibodies, and 1 MERS-CoV–positive serum sample was weakly positive for N protein antibodies (Figure 3, panels C, D). We also detected seroconversion among the 3 patients with COVID-19. Because serum samples from the 3 patients were collected at a limited number of time points, it was difficult to accurately assess time for seroconversion. To accurately assess time of seroconversion, a larger number of longitudinal samples is needed. Overall, these validated ELISAs for different antigens can be useful for epidemiologic studies and for evaluation of vaccine-induced immune responses.

**Figure 4:**
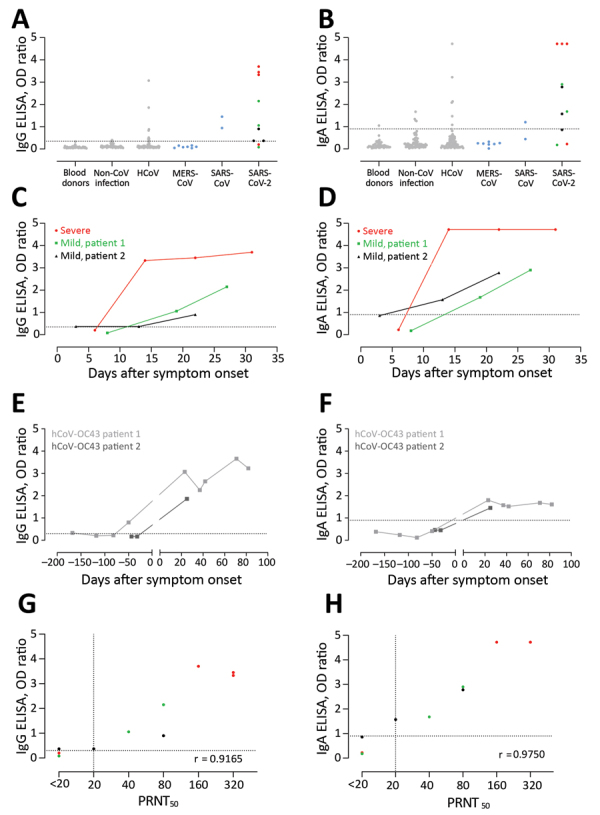
Validation of 2 commercial ELISAs for detection of SARS-CoV-2–specific IgG (A, C, E, G) and IgA (B, D, F, H). A, B) Validation of the specificity of the 2 ELISA platforms; C, D) kinetics of antibody responses in 3 COVID-19 patients; E, F) cross-reactivity of HCoV-OC43 serum samples in commercial platforms; G, H) correlation between antibody responses detected by the ELISAs and the plaque reduction neutralization assay. Gray dots indicate specificity cohorts A–C, including healthy blood donors (n = 45), non-CoV respiratory infections (n = 76), and HCoV infections (n = 75); blue dots indicate non-SARS-CoV-2 zoonotic coronavirus infections (i.e., MERS-CoV [n = 7] and SARS-CoV [n = 2]); red dots indicate patients with severe COVID-19; and green and black dots indicate patients with mild COVID-19. Dotted horizontal lines indicate ELISA cutoff values. CoV, coronavirus; COVID-19, coronavirus disease 2019; HCoV, human coronavirus; MERS-CoV, Middle East respiratory syndrome coronavirus; N, nucleocapsid; OD, optical density; PRNT_50_, plaque reduction neutralization assay; RBD, receptor-binding domain; RFU, relative fluorescence unit; S, spike; SARS-CoV-2; severe acute respiratory syndrome coronavirus 2.

Next, we validated the sensitivity and specificity of 2 commercial ELISA kits for detecting S1-specific IgG and IgA by using the same cohort (Table 1; Figure 4). All 3 COVID-19 patients had reactive antibodies detected by the IgG (6/10 serum samples) and IgA (7/10 serum samples) ELISAs (Figure 4). We also detected reactivity of serum samples from the validation cohorts A–D; 11/203 for IgA and 8/203 for IgG ELISAs. Serum samples from 2 patients infected with HCoV-OC43 (a betacoronavirus) were reactive in both IgG and IgA ELISA kits. We have reported the cross-reactivity of these serum samples in a MERS-CoV S1 IgG ELISA kit (*6*). We confirmed the cross-reactivity of the 2 serum samples by testing 12 serum samples from both patients that were collected at different time points (pre-OC43 and post-OC43 infection). Although all preinfection serum samples were negative, all postinfection serum samples were reactive in the IgG and IgA ELISAs. 

**Figure 5:**
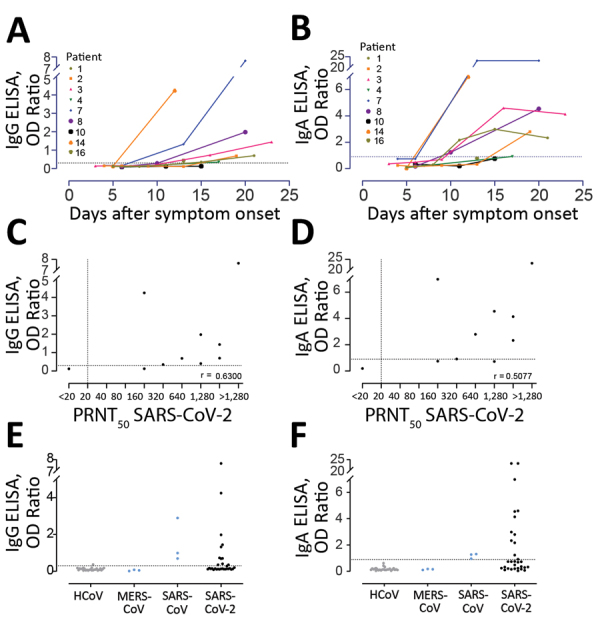
Sensitivity of 2 commercial ELISAs for detection of SARS-CoV-2 specific IgG (A, C, E) and IgA (B, D, F). A, B) Kinetics of antibody responses in 9 COVID-19 patients from Germany; C, D) correlation between antibody responses detected by the ELISAs and the plaque reduction neutralization assay; E, F) kits were tested for specificity by using 18 serum samples from patients infected with HCoV (4 from patients infected with HCoV-229E, 3 from patients infected with HCoV-HKU1, 4 from patients infected with HCoV-NL63, and 7 from patients infected with HCoV-OC43), MERS-CoV (n = 3), and SARS-CoV (n = 3). Dotted horizontal lines indicate ELISA cutoff values. COVID-19, coronavirus disease 2019; HCoV, human coronavirus; MERS-CoV, Middle East respiratory syndrome coronavirus; N, nucleocapsid; OD, optical density; PRNT_50_, plaque reduction neutralization assay; RBD, receptor-binding domain; RFU, relative fluorescence unit; S, spike; SARS-CoV, severe acute respiratory syndrome coronavirus; SARS-CoV-2; severe acute respiratory syndrome coronavirus 2.

Further validation was also made in a different laboratory by using 31 serum samples collected from 9 COVID-19 patients in Germany (R. Wölfel et al., unpub. data, https://doi.org/10.1101/2020.03.05.20030502) at different time points (3–23 days after disease onset); a specificity cohort composed of 18 serum samples from persons infected with HCoV (4 samples from persons infected with HCoV-229E, 3 from persons infected with HCoV-HKU1, 4 from persons infected with HCoV-NL63, and 7 from persons infected with HCoV-OC43); and 3 serum samples from persons infected with MERS-CoV and 3 samples from persons infected with SARS-CoV whose samples were collected 4–56 days after onset of disease onset (Figure 5). All 9 COVID-19 patients were previously confirmed to seroconvert at days 6–15 after onset of disease by use of a recombinant immunofluorescence test and PRNT. A total of 8/9 seroconverted patients showed reactivity above the implemented cutoff values in the IgG and IgA ELISA. A serum sample from 1 patient (Figure 5, panels A, B) had an antibody level slightly below the cutoff value, which might be explained by an overall reduced antibody response of this patient (PRNT_90_ = 10). Overall, the IgA-based ELISA kit was more sensitive but less specific than the IgG-based ELISA kit.

Finally, we compared the performance of different ELISAs for detection of antibodies among PCR-confirmed COVID-19 patients with that of PRNT, which is the standard for coronavirus serologic analysis (Tables 3, 4). The PRNT_50_ correlated strongly with different ELISAs; the commercial IgA ELISA showed the strongest correlation, followed by the S and N ELISA, which indicated their capacity to detect SARS-CoV-2–specific antibodies. However, a larger patient cohort is needed to assess the sensitivities of these platforms.

**Table 3:** Correlations between ODs/OD ratios and PRNT results of 10 serum samples obtained from 3 PCR-confirmed COVID-19 patients from France and tested in Rotterdam, the Netherlands, 6–27 d after diagnosis*

Test, virus	Correlations	In-house ELISAs						Euroimmun ELISAs	
		S1	N	RBD	S	S1A		IgA	IgG
PRNT_50_, SARS-CoV-2	Spearman ρ value	0.87	0.94	0.92	0.94	0.93		0.98	0.92
	2-tailed p value	0.0021	0.0002	0.0005	0.0002	0.0003		<0.0001	0.0005
	p value summary	<0.01	<0.001	<0.001	<0.001	<0.001		<0.0001	<0.001
PRNT_90_, SARS-CoV-2	Spearman ρ value	0.88	0.88	0.88	0.88	0.88		0.93	0.88
	2-tailed p value	0.0024	0.0024	0.0024	0.0024	0.0024		0.0008	0.002
	p value summary	<0.01	<0.01	<0.01	<0.01	<0.01		<0.001	<0.01

*COVID-19, coronavirus disease 2019; OD, optical density; N, nucleocapsid; PRNT_50_, 50% plaque reduction neutralization test; PRNT_90_, 90% plaque reduction neutralization test; RBD, receptor-binding domain; S, spike; SARS-CoV-2, severe acute respiratory syndrome coronavirus 2.

**Table 4:** Correlations between ODs/OD ratios and PRNT results of 31 serum samples from 9 PCR-confirmed COVID-19 patients tested from Germany and tested in in Berlin, Germany, 3–23 d after disease onset*

Test, virus	Correlation	Euroimmun ELISAs	
		IgA	IgG
PRNT_50_, SARS-CoV-2	Spearman ρ value	0.63	0.5077
	2-tailed p value	0.056	0.1368
	p value summary	NS	NS
PRNT_90_, SARS-CoV-2	Spearman ρ value	0.7922	0.8525
	2-tailed p value	0.0004	<0.0001
	p value summary	<0.001	<0.0001

*COVID-19, coronavirus disease 2019; NS, not significant; OD, optical density; PRNT_50_, 50% plaque reduction neutralization test; PRNT_90_, 90% plaque reduction neutralization test; SARS-CoV-2, severe acute respiratory syndrome coronavirus 2.

## Discussion

Validated SARS-CoV-2 serologic assays are urgently needed for contact tracing, epidemiologic and vaccine evaluation studies. Because the N and S proteins are the main immunogenic coronavirus proteins, we developed ELISA-based assays that were able to detect antibodies to these 2 proteins, and to the 2 S domains, S1^A^, and RBD. Results for these assays correlated strongly with results of the PRNT_50_. Because most humans have antibodies against the 4 endemic human coronaviruses, it was crucial to verify the specificity of these assays to avoid false-positive results. In addition, the 2 zoonotic coronaviruses, SARS-CoV and MERS-CoV, are also betacoronaviruses, increasing the potential for cross-reactivity. Among the S antigens tested, S1 was more specific than S in detecting SARS-CoV-2 antibodies, as MERS-CoV S cross-reactive antibodies were detected in serum of 1 of the COVID-19 patients, which was not seen when MERS-CoV S1 was used for testing. This finding could be explained by the high degree of conservation in the coronavirus S2 subunit relative to S1 (Table 2). Therefore, consistent with our earlier findings for serologic analysis of MERS-CoV (*6*), S1 is a specific antigen for SARS-CoV-2 diagnostics.

When testing the specificity of S1 or its RBD for detecting SARS-CoV-2 antibodies, none of the serum samples from the validation cohorts (A–E) showed any reactivity, except for serum samples from patients with SARS-CoV. This finding is not unexpected because cross-reactivity resulted from the high degree of similarity between S1 and RBD of SARS-CoV and SARS-CoV-2 (Table 2). However, SARS-CoV has not circulated in the human population since 2003 (i.e., 17 years ago), and an earlier study reported waning of SARS-CoV–specific antibodies, which made them undetectable in 21 (91%) of 23 serum samples tested 6 years after infection (*11*). It is therefore unlikely that antibodies to this virus are present in the population, and thus it is unlikely that false-positives results are caused by reactivity of SARS-CoV antibodies.

We used the high degree of similarity between the SARS-CoV and SARS-CoV-2 proteins to develop a new in-house N protein ELISA, in which we used SARS-CoV N protein (90% similar to SARS-CoV-2 N protein) as antigen. The N protein ELISA could detect SARS-CoV-2–specific antibodies with high specificity and sensitivity. Using the 3 different validated ELISAs, we found that antibody levels were higher after severe infection than after mild infections; similar findings have been reported earlier for MERS-CoV (*12*,*13*). However, this finding needs to be confirmed in a larger cohort of patients with various degrees of disease severity, and it highlights the potential need for a sensitive assay to avoid missing persons who have milder infections in epidemiologic studies.

In addition, IgG seroconversion can be reliably confirmed in the second week after disease onset. However, because of the limited number of longitudinal serum samples from COVID-19 patients tested by the in-house assays, it was difficult to accurately assess time for seroconversion. For this assessment, a larger number of longitudinal samples is needed. In the 3 in-house ELISAs tested, the RBD and N protein ELISAs were more sensitive than S1 ELISA in detecting antibodies in mildly infected patients and showed stronger correlations with PRNT_50_ titers. Therefore, detecting antibodies against 2 different antigens might be needed to confirm the findings and avoid false-negative results in surveillance studies. However, the sensitivities of the assays need to be further validated with a larger cohort.

We validated β-versions of IgA and S1 IgG commercial ELISAs in 2 different laboratories. The IgA-based ELISA showed higher sensitivity than the IgG-based ELISA, whereas the IgG ELISA showed higher specificity than the IgA ELISA. Although the IgA and IgG assays can be used for serologic diagnosis, IgG is longer lived (*14*) and thus is preferred for serosurveillance studies. We observed some cross-reactivity in both ELISAs with serum samples from the same 2 HCoV-OC43 patients in which these samples showed cross-reactivity in a MERS-CoV S1 IgG ELISA (*6*) despite the different antigen used. This finding indicates a response to another protein that could be in the blocking or coating matrix, apart from the specific antigen coated, resulting in this consistent false-positive result.

Overall, the assays developed and validated in this study could be instrumental for patient contact tracing, serosurveillance studies, and vaccine evaluation studies. However, because various studies will be conducted in different laboratories, it is crucial to calibrate and standardize assays developed by different laboratories by using well-defined standard references as part of diagnostic assay validation. This standardization is not only needed to reduce interassay variability, but to also correlate results obtained from different laboratories that use various assays (*15*). This correlation is crucial for better comparison and interpretation of results from different studies; evaluating vaccine trials; enabling uniform assessment of immunogenicity, efficacy; and better understanding of correlates of immune protection (*16*). Thus, setting up reference panels is a vital element in our preparedness approaches to emerging viruses.
